# Using the Eucalyptus genome to understand the evolution of plant secondary metabolites in the Myrtaceae

**DOI:** 10.1186/1753-6561-5-S7-O11

**Published:** 2011-09-13

**Authors:** Carsten Kulheim, Hamish Webb, Suat Hui Yeoh, Ian Wallis, Gavin Moran, William Foley

**Affiliations:** 1 Australian National University, Australia

## Background

*Eucalyptus* trees (family Myrtaceae) are well-known for their high foliar content of several classes of secondary metabolites and these have a strong effect on the feeding patterns of several species of marsupials and at least some insects. Best known are the essential oils, which is mostly a mixture of terpenoids, but there are also significant concentrations of flavonoid and formylated phloroglucinol compounds. There is extensive quantitative and qualitative variation within and between species of Myrtaceae in these chemical groups and all appear to be under strong genetic control with heritabilities (*H^2^*) between 0.3 and 0.9. As well as being important ecologically, the terpenes in particular are valued as industrial and medicinal products and Australia supports a strong essential oil industry focused on *Eucalyptus* and *Melaleuca* foliar oils.

## Results and discussion

The *Eucalyptus grandis* genome provides the opportunity to discover the genetic makeup of the biosynthetic pathways for secondary metabolites. We present data from pathways leading into the biosynthesis of terpenes, flavonoids and lignins. The homology of genes and gene families were investigated and compared to a variety of other species including poplar (*Populus trichocarpa*), grape (*Vitis vinifera*) and apple (*Malus* x *domesticus*). For example, terpene synthases (the gene family responsible for the final step in the terpene biosynthesis) has 120 members in the genome of *Eucalyptus grandis*, compared to 44 and 99 in poplar and grape, respectively (Table 1). Genes of the biosynthetic pathways for secondary metabolites were mapped to the *Eucalyptus grandis* genome and their location was compared to a number of quantitative trait loci (QTL) studies that investigated variability in secondary metabolites and wood properties in eucalypts. This approach allowed the discovery of candidate genes for a large number of QTL.

**Table 1:** Number of TPS loci in annotated genomes and putative loci in *E. grandis*

	*A. thaliana*	*P. trichocarpa*	*O. sativa*	*V. vinifera*	*E. grandis*
TPS-a (sesqui)	23	21	31	42	53
TPS-b (mono)	6	13	0	30	38
TPS-b (hemi)	0	3	0	3	11
TPS-c, -e, -f	3	5	13	8	2
TPS-g (mono)	1	2	2	16	16
Sum of TPS genes	33	44	46	99	120

Understanding the genetic basis of variations in quantitative traits provides insights into ecosystem function and at the same time may help breeders in the essential oil industry. We have characterized trait associations with polymorphisms from *Eucalyptus globulus*, investigating 200 SNPs and roughly 40 traits ranging from terpenoids to terpene-adducts to flavonoids and to tannin-related traits. We discovered several significant trait associations between allelic variants in the chloroplastic MEP pathway and monoterpenes and between the cytosolic MVA pathway and sesquiterpenes, as well as one allelic variant in a prenyl pyrophosphate synthase that associates with the ratio of monoterpenes to sesquiterpenes. Loci with significant associations were mapped to the *Eucalyptus grandis* genome and compared to published QTL datasets that investigated similar traits. These results represent the first species wide analysis of the molecular basis of quantitative variation in secondary metabolites in any tree.

## Conclusions

The publicly available genome sequence of Eucalyptus grandis is a great resource that can be applied to a variety of questions including the genetic make-up of gene families for biosynthesis of plant secondary metabolites, genome organization of these genes and evolution of traits such as resistance to herbivores or the ability to re-sprout after fire. Combining studies of association genetics, QTL studies together with the genome sequence helps to shed light on the underlying control mechanisms of phenotypic variation.

